# Patient Design: The Importance of Including Patients in Designing Health Care

**DOI:** 10.2196/39178

**Published:** 2022-08-31

**Authors:** Bertalan Meskó, Dave deBronkart

**Affiliations:** 1 The Medical Futurist Institute Budapest Hungary; 2 Department of Behavioural Sciences Semmelweis University Budapest Hungary; 3 e-Patient Dave, LLC Boston, MA United States; 4 Society for Participatory Medicine Nutting Lake, MA United States

**Keywords:** patient, patient design, user design, patient centric, patient focus, digital health, future, empowerment, involvement, participatory, engagement, participation, patient centred, patient centered

## Abstract

A paradigm shift is underway in the patient-clinician relationship, driven by irreversible changes in information access, yet the model under which clinicians are trained, care is conducted, and care delivery is designed has not changed significantly even though we call it “patient centered.” Humanity endured centuries in which even doctors had little idea what the patient’s problem really was. Science slowly solved that, and for a century, only doctors could know what was worth knowing. Today, the rise of the internet and digital health has led to the end of that era. We are already witnessing early signs of the era of participatory health: genuinely empowered people living their lives and managing their health according to their own priorities, in partnership and consultation with physicians as needed. This may feel like a threat to the physician’s sacred role, but it is no more so than when physicians adopted informed consent and then shared decision-making. In the 2010s, many pharmaceutical, medical, and health care companies started to use patient centricity as a mantra. We argue that to drive this paradigm change fully into existence, we need to shift “patient centricity” from a relatively passive process, driven by industry needs, into a far more active, collaborative process driven by both parties’ needs and preferences. To build this new world of practice and workflow, we simply must engage with patients as true partners. To achieve medicine’s new potential, it must be optimized around the wants and priorities of the ultimate stakeholder—the party that has the most at stake in how it all plays out: the patient. Patient design is the approach that can make it happen.

## The Short History of Patient Empowerment

Health care has been going through a paradigm shift in the 21st century, as per Thomas Kuhn’s 1962 classic, *The Structure of Scientific Revolutions* [1]. Kuhn was an American philosopher who was influential in both academic and popular circles, introducing the term “paradigm shift.” His “blockbuster” book claimed that sometimes a scientific field discovers it was wrong about something important.

Kuhn [1] wrote that “perhaps science does not develop by the accumulation of individual discoveries and inventions,” but that “discovery commences with the awareness of anomaly, i.e., with the recognition that nature has somehow violated the paradigm-induced expectations that govern normal science.”

We are at the point of detecting such anomalies in health care. For centuries, the dominant paradigm has been that patients do not and cannot contribute to their care, especially medical decisions concerning their case. However, as the ivory tower of medicine started breaking down in the early 21st century, empowered patients started bringing real value to their own cases, violating the paradigm-induced expectations that dictate the culture of medicine [1].

Kuhn [1] wrote that when too many anomalies accumulate, a field goes into crisis mode until a new paradigm is developed and accepted. We assert that the current paradigm cannot explain nor cope with the cluster of anomalies in which patients are genuinely creating value in health care, and to ignore this is to suboptimize health care in a way we can no longer afford.

Thus, this crisis stage has arrived, but as often happens, the causes of the anomalies are poorly understood, which leads to confusion. No new paradigm can arise, letting the field advance, while confusion reigns. Here, we present those causes—the factors that did not exist a generation ago, and now do:

- Consumerism: the cultural willingness of consumers to pursue their own priorities
- Information liquidity brought by the internet, which eradicated the belief that only people from the “priesthood” could know certain facts
- Advanced consumer health technologies putting unprecedented knowledge in the hands of consumers who had previously been uninformed
- Global supply chains making it possible for new products and technologies to reach patients worldwide
- The rise of social media enabling peer-to-peer communication among patients about needs and solutions

In truth, patient empowerment has been evolving for decades, but information liquidity and access to technology made it explode in this century—and become visible to the naked eye. At the time of his death in 2006, “Doc Tom” Ferguson, MD, was working on a white paper funded by the Robert Wood Johnson Foundation, “e-Patients: How They Can Help Heal Healthcare,” documenting what empowered and engaged patients had been doing as far back as the 1980s. His colleagues published the paper in 2007, and, in 2009, founded the Society for Participatory Medicine [2].

The cultural transformation we call digital health represents this paradigm shift and is a continuation of that vision [3].

## The Practical Reality of Patient Empowerment

Today, except for the commercial obstacle inserted by paywalls, patients can have access to the same online health care resources, studies, and data as medical professionals [4]. Empowered patients want to get engaged in their health or disease management. There are many examples of how patients take their lives into their own hands. From joining patient communities online to using a range of digital health sensors, they bring new value to the table. In doing so, they violate the paradigm’s cultural expectation that only doctors know anything useful [5].

Empowered patients also put pressure on regulators. The #WeAreNotWaiting movement is a community of patients with diabetes taking disease management into their own hands by organizing themselves and developing applications, platforms, and other solutions to help each other beat their disease. They even created the “DIY pancreas” software, which automatically provides patients with the right doses of insulin based on their blood glucose level [6]. The software was created entirely by the patient community with no contribution from medical professionals.

It should be noted that this OpenAPS (Open Artificial Pancreas System) software is the second most-forked item on all of GitHub (Microsoft Corp) because almost all patients tweak the code to suit their own biological response. In other words, the app is designed from the ground up to be fully configurable to suit individual needs. This is the most advanced example of patient design we have seen.

Patient scholars have published in prestigious medical journals [7-9]. The #PatientsIncluded movement has led to involving patients in medical events either as speakers or cohosts. Governments such as that of New Zealand have started developing digital health policies featuring empowered patients.

These examples further underscore that a more patient-inclusive design approach is already emerging and will inevitably be the norm. The only thing holding it back is cultural resistance, which is why we say digital health is a cultural transformation.

## The Rise of Patient Centricity and Patient Design

In the 2010s, myriad pharmaceutical, medical, and health care companies started to use patient centricity as a mantra. Each claimed that their company is patient centric and thus ahead of the others. Pharmaceutical company executives started making “putting patients first” part of their slogans and internal documents. A 2020 survey revealed that 85% of companies were raising their investment in patient-centric capabilities over the next 18 months [10].

Patients want more reliable and relatable health information from the companies that make their medication, so this was an obvious step forward for the industry. A 2019 survey indicated that 76% of patients expect pharmaceutical organizations to provide them with tools and support services [11].

At the same time, policy makers started adopting this theme too. The US Food and Drug Administration (FDA) launched the Patient Engagement Advisory Committee in 2017. The committee provides advice to the FDA commissioner or designee on complex issues relating to medical devices, the regulation of devices, and their use by patients.

## The Need for a New Level of Patient Centricity: Patient Design

To drive this paradigm change fully into existence, we call for changing patient centricity from a relatively passive process, driven by industry needs, into a far more active, collaborative process driven by both parties’ needs and preferences. In short, it is no longer viable for patient centricity to mean, “We were *thinking about you* while *we* made *our* decisions.”

From the patient’s perspective, patient centricity has been a passive process since the inclusion of their opinion in the final design depends solely on those who invite the patient's opinion. That approach may sound patient centric, but in this scenario, patients’ voices literally have no power since all decisions are still made by the project organizers. Sociologist Sherry Arnstein bluntly called this tokenism [12].

In contrast to this, patient design means patients are involved in the highest level of decision-making in the organization, essentially having patients advise the chief executive officer of a company or the head of a health care organization.

Patient design is a so-called “co-design” approach. Co-design is defined as “a creative practice that can be used to improve customer experience and enhance value” [13]. The approach involves a wide range of people who are the experts of their experience and therefore make creative contributions that come from the perspective of the person who has the need. This is only possible by admitting to ourselves that the cared-for person just might know what they want and be well-informed! On what basis would someone assert otherwise?

The short-term benefits of such a co-design approach could include:

- More original ideas arising from more diverse perspectives and priorities
- Better achievement of consumer value (by incorporating the voice of the person for whom the project exists)
- Improved knowledge of patient needs
- Immediate validation of ideas
- More efficient decision-making
- Reduced development time
- Generally better cooperation between patients and companies or organizations.

The long-term benefits of such a co-design approach could include higher degrees of patient satisfaction, increased levels of support and enthusiasm for innovation, and a better relationship between patients and companies (Table 1).

**Table 1:** Comparison of patients’ role and power in token patient centricity versus patients as empowered design partners.

	Token “patient centricity”	Patients as design partners
Involvement of patients	Passive; when asked by the power holders	Active at all times
Patients’ decision power	None; their opinions are sought but need not be heeded. The system is free to continue not responding	Shared
Type of input provided by patients	Share their opinions when invited	Actively influence design decisions, including what gets worked on
Mode of involvement	Through surveys, questionnaires, and focus groups, all organized by the power holders	By sitting on project committees and advisory boards that set agendas
What level of decision-making patients influence	Any level within the organization	The highest level of decision-making

## Real-life Practical Examples of How Patient Designs Work

This social movement has already progressed to where examples exist to illustrate the shift in thinking—the paradigm change.

### Physical Products

For physical products, the Patient Innovation website [14] shares innovations developed by patients. Some focus on a disease, some are just for a symptom, while others enable a particular activity. What they have in common is that they all feature patient-centered thinking: they are expressions of what patients *want* to improve.

### Research

In research, patient voices are calling for researchers to change priorities to match patients’ urgent needs. The father of a son with suicide ideation told Dr Thomas Insel after a speech [15], “Our house is on fire, and you’re telling us what you learned about the chemistry of the paint.” The scientific literature may contain volumes about “the paint,” but Insel realized “this gap between our scientific progress and our public health failure.” He left academia to pursue product development to solve real-world problems.

The urgency articulated so powerfully by Insel’s audience member precisely echoes the urgency of AIDS activists in the last century who demanded that science respond more to patients’ immediate needs, not just long-term science.

When the husband of Bettina Ryll, MD, PhD, was dying of melanoma, she switched hats and observed the clinical trials process as a family stakeholder and was incensed to discover that researchers chose their work priorities without consulting the people who were dying. Today, she advocates in *Nature* for researchers to do just that [16].

### Clinical Design

In clinical design, the Oral and Maxillofacial Surgery Department of Radboud University Medical Centre Nijmegen in the Netherlands redesigned the whole department’s rooms based on advice from patients. This involved changes that enabled a more balanced patient-physician relationship and a comfortable atmosphere. They prioritized round tables over square ones for more friendly conversations and suggested brightly lit rooms with warm colors.

These examples merely illustrate how differently things can play out when, in fact, the patient truly has agency in influencing the nature of care.

While it may seem that we portray patient design in an almost utopian manner, we must note that such transitions—like all cultural changes—are time-consuming and complicated. An analogy is Title IX, a law introduced in the United States in 1972 that required schools to provide sports for girls, not just boys. It took a generation before the US women’s soccer team won the World Cup in 1991. A major consequence of an incorrect paradigm is that if we do not let women onto the pitch (or patients into the executive world), we have no chance of discovering their actual suppressed potential.

## Conclusion

We are at the end of the only period in history where physicians knew important scientific facts and medical insights that patients could not. For health care to achieve its potential in this new era, our methods, along with our paradigm, must change.

Before 1900, medical practice mostly lacked any scientific basis. Doctors were not even exposed to patients in medical school (patients were merely called “clinical material” in the Flexner Report!). Yet by the end of the 20th century, the internet let knowledge flow.

Now in the 21st century, a plethora of personal health devices gives patients access to *more* information than their physicians have. The possibility of true participatory medicine is on the horizon—patients with increasing autonomy living their lives according to their own priorities, in partnership and consultation with physicians *as needed*.

To build this new world of practice and workflow, we simply must engage with patients as true partners. To achieve medicine’s new potential, it must be optimized around the wants and priorities of the ultimate stakeholder—the party that has the most at stake in how it all plays out: the patient. Patient design is the approach that can make it happen.

## Abbreviations

OpenAPS: Open Artificial Pancreas System
FDA: US Food and Drug Administration
